# Trichomoniasis and associated co-infections of the genital tract among pregnant women presenting at two hospitals in Ghana

**DOI:** 10.1186/s12905-017-0489-5

**Published:** 2017-12-13

**Authors:** Richard H. Asmah, Harriet N. A. Blankson, Kekeli A. Seanefu, Noah Obeng-Nkrumah, Georgina Awuah-Mensah, Momodou Cham, Patrick F. Ayeh-Kumi

**Affiliations:** 1 Department of Medical Laboratory Sciences, School of Biomedical and Allied Health Sciences, College of Health Sciences, University of Ghana, Accra, Ghana; 2 Comboni Catholic Hospital, Sogakope, Ghana; 3 Department of Microbiology, School of Biomedical and Allied Health Sciences, College of Health Sciences, University of Ghana, Accra, Ghana

**Keywords:** *Trichomonas vaginalis*, Coinfections, *Gonococci*, *Proteus*, Pregnant women

## Abstract

**Background:**

*Trichomonas vaginalis* (*TV*) infection is the most prevalent non-viral sexually transmitted pathogen worldwide. Among pregnant women, the infection may cause adverse birth outcomes such as premature rupture of membranes and premature labour. In view of the paucity of information relating to *TV* among Ghanaian pregnant women, this study investigated its prevalence and associated co-infections among pregnant women.

**Methods:**

High vaginal swabs were obtained from 99 pregnant women using sterile cotton swab sticks. Wet preparation, Grams staining, culturing, coagulase and sensitivity testing were carried out to determine the presence of *TV* and associated microorganisms.

**Results:**

The prevalence *of TV* among the pregnant women was found to be 20.2% (*n* = 20). Concurring with Trichomoniasis, 75% (*n* = 15) of participants had other infections such as *Candida* with prevalence of 53% (*n* = 8), *Proteus* infection - 20% (*n* = 3), *Streptococcus* infection - 13% (*n* = 2) and other GNRs and *Gonococci* having 7% each (*n* = 1). Moreover, there was 86.9% (*n* = 86) prevalence of *Staphylococcus spp.* among study participants. There was statistically significant correlation between *TV* and *Gonococci* infection at a correlation co-efficient of 0.107 (*P* < 0.05) as well as significant correlation between *TV* and *Proteus spp*. at a correlation co-efficient of 0.189 (*P* < 0.05). *TV* infection was high (60%) among the most sexually active age group (19 to 29 yrs).

**Conclusion:**

There was 20.2% prevalence of *TV* among the pregnant women presenting at the hospitals, with *Gonococci* and *Proteus* infections being statistically significant associated infections.

## Background

*Trichomonas vaginalis (TV)* infection is the most prevalent non-viral Sexually Transmitted Infection (STI) globally; the World Health Organization (WHO) estimated prevalence from 170 million to 190 million cases worldwide yearly [1]. Sutton et al. (2007) reported a 13.3% prevalence in African American women with 85% of women found to have trichomoniasis presenting with no symptoms [2]. Although rate of STIs are decreasing, trichomoniasis however remains a very common infection [3].

Trichomoniasis infection has been associated with vaginitis, cervicitis, urethritis, pelvic inflammatory disease (PID), adverse birth outcomes [4] as well as increased transmission of HIV [5]. Though the disease can be easily treated and is preventable, it is often asymptomatic, and its acquisition and transmission are often accompanied by other serious STIs [6]. The fact that the infection may be asymptomatic makes it not only a personal problem, but also a public health challenge.

Poor sexual practices such as multiple partners and bad hygiene regarding reproductive organs increase incidence of vaginal trichomoniasis. Individuals of low socioeconomic status as well as those infected with HIV and Hepatitis B Virus are part of the at risk group [7].

A report by the JSI Research and Training Institute, Inc. (2013) indicates that populations of the urban centres in Ghana are better educated, have better access to health facilities are more exposed to health control messages than those in the rural communities [8]. Though studies have been conducted to ascertain the prevalence of *TV* infection in many countries, not much has not been recorded in Ghana especially on pregnant women. The study was thus conducted to determine the prevalence of this infection and any other genital infections among pregnant women presenting at two (2) major hospitals at Sogakope, a town in the Volta Region of Ghana.

## Methods

### Study area

This study was conducted in Sogakope at the Saint Comboni and the South Tongu District hospitals. Sogakope is a town in the South Tongu District, a district in the Volta Region of Ghana. It has a population of 104,194.

### Study design and sampling method

This research was designed as a facility-based cross-sectional study and it was conducted from April to June, 2016. Simple Random sampling was used.

### Study participants, inclusion and exclusion criteria

The participants of the study comprised pregnant women who had been received for antenatal care as well as those visiting the laboratory for routine urine examination at the Saint Comboni and the South Tongu District hospitals in Sogakope. Pregnant women in any of the trimesters who consented were allowed to participate in the study irrespective of age. Only participants who were permanent residents or have lived at Sogakope for at least 6 months were included in the research. Those excluded from the study were pregnant women who are on antibiotics and vaginal medications and pregnant women who had sex three days prior to data collection.

### Data collection procedure

Sociodemographic and medical history data were collected using structured questionnaires by engaging in a face to face interview with each consenting participant. Two sterile cotton tipped swab sticks, one after the other were inserted at least 2.5 cm (1 in.) into the vagina of each pregnant woman and turned in the inner wall while counting to 10 to collect vaginal fluid (secretions) onto the swab by a clinician. Informed consent was obtained from all participants (consent from parent or legal guardian was obtained from those below the age of 16).

### Laboratory analysis and diagnosis

One of the swabs taken was immediately washed in a drop of physiological saline on a clean grease-free labelled frosted end glass slide and cover slipped. Slides were scanned with ×10 objective and examined with ×40 objective using a light microscope. The second swab stick was stored in swab tubes containing Amies medium and transported to the microbiology lab of the SBAHS for culture and gram staining to identify any bacterial infections.

The procedure and principle for gram staining employed in the study as well as the agar preparation protocols involved were adopted from the book: *District Laboratory Practice in Tropical Countries* (Part II) by Cheesbrough (2005).

Prepared chocolate agar media were dried in an oven, labelled and made ready for inoculation. The swabs (in the transport medium) were used to make a pool each on a plate and subsequently streaked with a sterile inoculating loop after which they were incubated for 18 to 24 h.

### Data analysis

Data were entered into Statistical Package for Social Sciences (SPSS) version 20.0 and analyzed alongside with Microsoft excel 2013. Descriptive statistics such as bar and pie charts were used as well as Pearson correlation were used in describing the data. Data entered in the computer were locked with a password while other materials in hard copy were taken to the SBAHS archives.

## Results

A total of 99 pregnant women consented to participate in the study. They were placed into 3 groups. Those ages 18 years and below were (23), those between the ages 19 to 29 (42) and then those 30 years and above (34).

### Genital infections among study participants

**Fig. 1:**
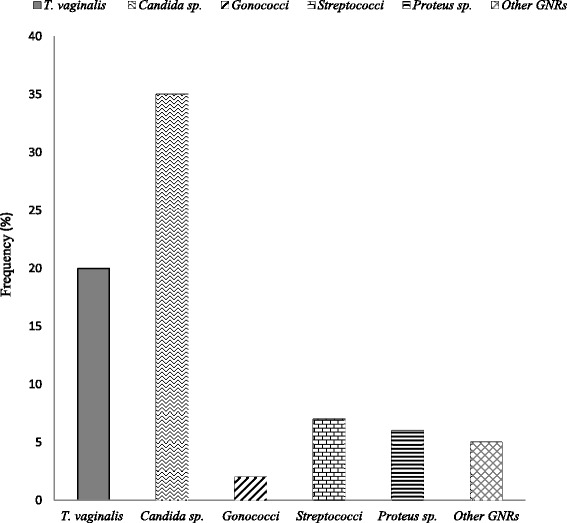
Genital Infections among Study Participants, GNRs = Gram Negative Rods

The prevalence of *TV* was found to be 20.2% which corresponds to twenty (20) of the overall participants in the research study (Fig. 1).

Several other infections were identified among the study participants. These infections and their frequencies presented in Fig. 3 show *Candida* to be the highest infection (*n* = 35; 35.4%) recorded.

**Fig. 2:**
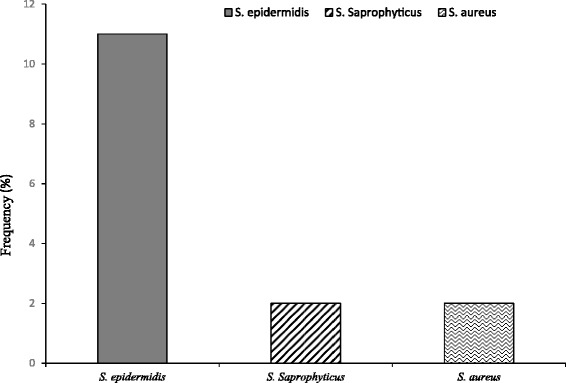
Distribution of *Staphylococcus* species Among *TV* Positives

It is noteworthy that 86 out of the 99 participants (86.9%) were positive for *Staphylococcus spp.* Similarly, out of the positive *TV* cases (*n* = 20), fifteen (15) were *Staph* positive. Species identification of the *Staphs* among participants with *T. vaginalis* indicated however that 73.4% (*n* = 11) are *S. epidermidis,* a normal flora of the skin as shown in Fig. 2.

Table 1 reveals the bacteria identification criteria using gram staining and biochemical (coagulase) and Sensitivity tests using Novobiocin antibiotic.

**Table 1:** Bacteria identified based on biochemical reactions

Gram Stain Reaction	Coagulase Test	Swarming on Chocolate Media	Susceptibility to Novobiocin	Bacteria identified
Gram negative rod	NA	Positive	NA	*Proteus sp*
Gram negative rod	NA	NA	NA	Other GN Rods
Gram positive cocci in clusters	Positive	NA	NA	*Staphylococcus aureus*
Gram positive cocci in clusters	Negative	NA	Resistant	*Staphylococcus saprophyticus*
Gram positive cocci in clusters	Negative	NA	Susceptible	*Staphylococcus epidermidis*
Gram positive cocci in chains	NA	NA	NA	*Streptococcus sp.*

*NA*: Not Applicable

### Trichomoniasis with other genital infections

**Fig. 3:**
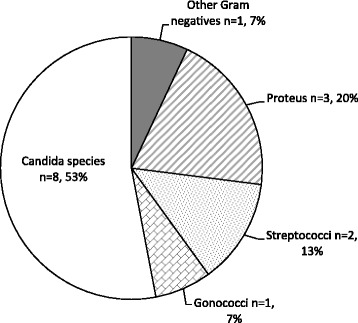
Co-infections with *TV*

One major objective of the study was to identify genital infections that co-existed with *TV.* Figure 3 below reveals that *Candida* (53%) co-existed more with *T. vaginalis.* Other infections recorded were as low as 7% (other GNRs).

### Association between *TV* and other genital infections

Statistical correlation was done for four (4) organisms (*Proteu*s, *Strep*, *Candida*, and *Gonococci*) that were found to co-exist with *TV*. Tables 2 and 3 show the findings.

**Table 2:** Correlation between *TV*, *Candida* and *Gonoccoci* infection

		*Candida* infection	*Gonococci* infection	*T. vaginalis* infection
*Candida* infection	Pearson Correlation	1	−.106	.049^b^
	Sig. (2-tailed)		.296	.631
	N	99	99	99
*Gonococci* infection	Pearson Correlation	−.106	1	.107^a^
	Sig. (2-tailed)	.296		.294
	N	99	99	99
*TV* infection	Pearson Correlation	.049^b^	.107^a^	1
	Sig. (2-tailed)	.631	.294	
	N	99	99	99

^a^Correlation is significant at the 0.05 level (2-tailed)

^b^Correlation is significant at the 0.01 level (2-tailed)

**Table 3:** Correlations between *TV*, *Strep* and *Proteus*

		*Streptococci* species	*Proteus* species	*Trichomonas vaginalis* infection
*Streptococci* species	Pearson Correlation	1	−.070	.057^b^
	Sig. (2-tailed)		.491	.572
	N	99	99	99
*Proteus* species	Pearson Correlation	−.070	1	.189^a^
	Sig. (2-tailed)	.491		.062
	N	99	99	99
*Trichomonas vaginalis* infection	Pearson Correlation	.057^b^	.189^a^	1
	Sig. (2-tailed)	.572	.062	
	N	99	99	99

^a^Correlation is significant at the 0.05 level (2-tailed)

^b^Correlation is significant at the 0.01 level (2-tailed)

The analyses showed that *TV* and *Candida* had a positive correlation that was statistically significant (0.049, *p* = 0.05), as shown in Table 2 (confidence interval 99%). There was also a statistically significant correlation between *TV* and *Gonococci* infection at a correlation co-efficient of 0.107 (*P*-value = 0.05).

The correlation matrix (Table 3) also points out a significant correlation between *TV and Proteus spp*. at a correlation co-efficient of 0.189 (*P*-value = 0.05). Similarly, at a correlation co-efficient of 0.057 (*P*-value =0.05), there was a statistically significant relation between *Strep spp*. and *TV* infection at a significant level of 0.01 (99% confidence interval).

### Age association with *TV* infection

The data also showed an association of *TV* infection with age based on the three groups formed (≤18, 19–29 years, 30 ≥). The findings captured in Table 4 reveal that the age group 19 to 29 years has the highest frequency (60%) of *TV* infection.

**Table 4:** Relationship between Age and *TV* infection among Participants

Age	Frequency	Percent	Cumulative Percent (%)
18 yrs. and below	3	15.0	15.0
19 to 29 yrs	12	60.0	75.0
30 yrs. and above	5	25.0	100.0
Total	20	100.0	

## Discussion

Trichomoniasis is a common infection of the genital tract caused by a flagellated protozoon*, Trichomonas vaginalis* and considered chiefly as sexually transmitted since non-veneral transmission has not yet been well documented or published [9] It may be asymptomatic [5] in a large proportion of infected women though it elicits an acute inflammatory response resulting in vaginal discharge, vaginal itching or irritation and a frothy grey to green- yellow discharge, vaginal malodor and dysuria. *TV* may lead to premature rupture of membranes, premature labour and low birth weight [10–12] which makes the infection a serious health concern among pregnant women.

### Prevalence of *T. vaginalis* among participants

Studies have been conducted to ascertain the prevalence of *TV* infection in many countries, however much has not been recorded in Ghana especially on pregnant women. Results from this study indicated that the prevalence of *TV* among pregnant women presenting at the South Tongu District and St. Comboni Hospitals in Sogakope is 20.2%.

It was also observed that there were several other genital infections among the study participants, namely *Candida spp*. (35.4%) *Streptococcus spp.* (7.1%) *Gonococcus spp.* (2.0%). *Proteus spp.* (6.1%) and other GNRs (5.1%).

The above notwithstanding, there was 86.9% (*n* = 86) prevalence of *Staphylococcus spp.* among the study participants. Further investigation was done especially on the positive *TV* cases that were also *Staph* positive (*n* = 15). It was revealed that 73.4% (*n* = 11) are *S. epidermidis* as shown in Fig. 2 with the rest being *S. aureus* (*n* = 2) and *S. saprophyticus* (*n* = 2). *S. epidermidis* has been regarded as typically normal flora of the skin and less commonly as mucosal flora that is non-pathogenic, controlling growth of the more pathogenic counterpart *S. aureus* [13]. Their isolation in the study could be due to contamination from labia majora of the vagina during sample collection. The bacteria *S. epidermidis* have recently been associated with nosocomial infections, where the organism is responsible for about 22% of bloodstream infections. It does not necessarily attack hosts by producing toxins but it may have immune evasion mechanisms which allows its proliferation [14]. Similarly, *S. saprophyticus* is a normal flora of the female genital tract and perineum and can cause 10–20% of urinary tract infections if displaced from the normal flora of the vagina and perineum into the urethra [15]. Care must hence be taken in such instances of its infection even though it does not demand much attention.

Among others, one mode of transmission of *S. aureus* is through direct contact with objects that are contaminated by the bacteria. It is opportunistic in such a way that it takes advantage of a breach in skin or other entry sites to become pathogenic [16]. Hence, infection of the female genital tract by *S. aureus* hence should be of great concern and all women need to be educated against it.

### Trichomoniasis with other genital infections

The prevalence of *TV* among the pregnant women who presented at the South Tongu District and St. Comboni Hospitals in Sogakope during the period of study was found to be 20.2% (*n* = 20). Co-ocurring with Trichomoniasis, fifteen (15) participants had infections such as *Gonorrhea, Candida spp., Streptococci spp., Proteus spp.* and other Gram negative rods as shown in Fig. 3. Candida infection tops the list with 53% (*n* = 8), followed by Proteus infection with 20% (*n* = 3), *Streptococcus* infection with 13% (n = 2) and other GNRs and *Gonococci* with 7% each (*n* = 1).

Yeast infections are a common type of vaginal infections and they are especially common in pregnant women as they become immunocompromised during that period [17]. It was therefore not surprising to find that *Candida spp.* was the leading infection that co-existed with *TV* among the study participants. *Proteus* species as well as other GNRs such as *Klebsiella spp., E. coli,* etc. are implicated as serious causes of infection in humans most commonly found urinary tract [18]*.* Urinary tract infections are the most common clinical manifestations of *Proteus* infections [19]. This could be due to the proximity of the female genitalia to the anal orifice which may encourage contamination/infection of the vagina with *Enterobacteriaceae* (GNRs) under poor hygienic conditions. There is therefore a need for women especially those in less health endowed communities and rural areas to be educated on the relevance of personal hygiene and the appropriate cleaning of the private part (anus and vagina).

According to the Gonorrhea CDC Fact Sheet (2014), “anyone who is sexually active can get gonorrhea” as STD. It was further stated that “a pregnant woman with gonorrhea can give the infection to her baby during childbirth”. The complications that come along with this infection such as neonatal conjunctivitis (ophthalmia neonatorum) should be given much attention in order to ameliorate or reduce the rate of incidence. Aside abstinence from all forms of sex, the fact sheet advices a mutually monogamous relationship with a known gonorrhea negative individual or protection with the use of condom [20].

### Correlation between *TV* infection and its associated infections

Of the diverse organisms that were found to co-exist with *TV*, there was analysis to identify any significant association or correlation as revealed in Tables 2 and 3.

*Trichomonas vaginalis* and *Candida* had a positive correlation that was statistically significant (0.049, *p* = 0.05) as shown in Table 2 (confidence interval 99%). Though this correlation is not significant as per the 95% confidence interval set, the correlation revealed a statistically significant co-efficient at the 99% confidence interval. This suggests that there is an association between *TV* infection and *Candida* infection though not a very strong one.

There was as well statistically significant correlation between *TV* and *Gonococci* infection at correlation co-efficient of 0.107 (*P*-value = 0.05) (Table 2). This portrays a strong relationship between the two infections. That one out of the two positive *Gonococci* cases encountered among the 99 participants was among the only 20 positive *TV* cases indicates that there is indeed a strong correlation between *TV* infection and *Gonococci* infection. Better still, there is a high probability for the two infections to co-exist since they are both STDs.

Additionally, the correlation matrix (Table 3) also points out a significant correlation between *TV* and *Proteus spp*. at a correlation co-efficient of 0.189 (*P*-value = 0.05). Similarly, at a correlation co-efficient of 0.057 (*P*-value =0.05), there was a statistically significant relation between *Strep spp*. and *TV* infection at a significant level of 0.01 (99% confidence interval). Per Edwards et al. (2016), “the pathology of trichomoniasis results from damage to epithelia, caused by a variety of processes during infection and that recent work has highlighted the complex interactions between parasite and host, commensal microbiome and accompanying symbionts” [21]. This explains why infections such as those caused by *Strep spp.* and *Proteus spp.* could be associated with *TV.* For example, in the condition of *TV* infection, the epithelia of the vagina may be disturbed alongside the normal flora of the site such as lactobacilli. Since these bacteria are the main organisms responsible for the normal pH of the vagina, their disturbance could pose a great threat where other opportunistic infections may set in. Similarly, co-infections may result in one becoming immunocompromised, less productive and incurring of much loss in the treatment of infections.

### Age relationship with *TV*

Participants of age group 19 to 29 years had the highest frequency of infection (60%). Those who were 30 and above had a 25% frequency while those who were 18 and below had the lowest frequency, 15%. The results correspond to the fact that sexually active individuals fall within the highest age group frequency and hence can be regarded as a sound reason for such results. According to the World Health Organization (WHO), *TV* infection is a sensitive marker of high-risk sexual behavior [22]. The findings also agree with a research by Nazari et al. (2015) conducted in Iran to identify the prevalence of *TV* among women. They found that infection rate was lower in the less than 20 yr. old groups (5.6%) and higher in the sexually active year groups (20 yr. to 40 yrs. – 62%) [12].

## Conclusion

*TV,* mostly an asymptomatic sexually transmitted infection, is predominantly evident in sexually active individuals and pregnant women fall into this category. The immunocompromising state of a pregnant woman is a contributing factor for the manifestation of infections which can have adverse effects on the mother and the unborn child. It is noteworthy that most *TV* infections among pregnant women are usually co-infections with other genital tract infections and their associated complications.

The rampant nature of the condition in recent times, despite all other strategies to curb it, indicates that it is still a serious public health concern that needs to be addressed. Pregnant women should particularly be encouraged to have themselves screened for the presence of the infection. Sexually active individuals ought to ensure faithfulness to their partners or where in doubt, maximize the use of protection in sexual intercourse to protect themselves against infection.

## Abbreviations

BV: Bacterial Vaginosis
DNA: Deoxyribonucleic Acid
GNRs: Gram Negative Rods
HBV: Hepatitis B Virus
HIV: Human Immunodeficiency Virus
NAAT: Nucleic Acid Amplification Technique
pH: Power of hydrogen
PID: Pelvic Inflammatory Disease
RTIs: Reproductive Tract Infections
SBAHS: School of Biomedical and Allied Health Sciences
St.: Saint
STI: Sexually Transmitted Infection
*TV*: *Trichomonas vaginalis*
US CDC: United State Centre for Disease Control
USAID: United States Aid for International Development
WHO: World Health Organization
Yrs: Years
